# First insights into the age of the giant ice deposits in the Eisriesenwelt cave (Austria)

**DOI:** 10.1038/s41598-024-61668-1

**Published:** 2024-05-14

**Authors:** Christoph Spötl, Jens Fohlmeister, Paula Reimer, Haiwei Zhang

**Affiliations:** 1 Institute of Geology, University of Innsbruck, 6020 Innsbruck, Austria; 2 German Federal Office for Radiation Protection, Koepenicker Allee 120, 10318 Berlin, Germany; 3 Centre for Climate, the Environment and Chronology (14CHRONO), School of Natural and Built Environment, Queen᾿S University Belfast, Belfast, BT7 1NN UK; 4 Institute of Global Environmental Change, Xi’an Jiaotong University, Xi’an, 710054 China

**Keywords:** Ice cave, Cryogenic cave carbonate, Radiocarbon, Holocene, Environmental sciences, Palaeoclimate, Climate sciences, Cryospheric science

## Abstract

Frozen water is the most widespread type of ice present in ice caves and forms ice stalagmites and stalactites as well as floor ice, which is often several meters thick. Organic macroremains are commonly rare in this type of cave ice, which makes it difficult to establish a chronology and severely limits the use of such ice deposits as paleoenvironmental archives. Here, the chronology of such ice deposits in the inner part of the glaciated Eisriesenwelt, one of the world’s largest ice caves located in the European Alps of Austria, is determined by a combination of radiocarbon and ^230^Th dating of cryogenic calcite. The data suggest that this cave ice has formed over the last three millennia, with a marked increase in the average accumulation rate during the thirteenth century, coinciding with the onset of the Little Ice Age in the Alps. Data from a second site closer to the entrance suggests that large parts of this tourist cave were likely ice-free during the Medieval Warm Period and that a substantial part of the ice is probably a relic of the Little Ice Age. The current warming has already penetrated deeper into the cave than during the Medieval Warm Period, although air exchange during the warm season is restricted by a door at the cave entrance.

## Introduction

The accumulation and long-term preservation of ice in caves is a phenomenon that is known from many karst regions and has been studied for more than a century^1,2^. Nevertheless, these so-called ice caves represent an little-studied part of the cryosphere and are threatened to disappear if global and regional temperatures continue to rise^3^.

There are two types of ice in ice caves: ice that has formed from snow that has fallen or slid into the caves and has become firn and finally ice through densification, freezing of infiltrating water, and refreezing of meltwater^4,5^. This type of ice is restricted to the near-entrance part of caves and is common in inclined or vertical caves lacking a lower entrance and hence acting as cold traps. A second type of ice is formed by the freezing of karst water entering the cave and is referred to as congelation ice. Its occurrence is not restricted to parts close to the entrance, but to cave parts with (seasonal) cooling due to specific air-flow patterns dictated by the cave geometry. In some ice caves (e.g., Dobšiná Ice Cave, Slovakia) congelation ice also formed not far from the entrance in a cold-trap setting. In the European Alps, this type of cave ice accumulated due to winter cooling of dynamically ventilated caves, controlled by the presence of multiple entrances at different elevations. Accumulations of firn and ice in (sub)vertical pits can reach several tens of meters in thickness, while congelation ice is usually no more than 10 m thick. The latter, however, can be laterally extensive and represents the largest ice caves on earth, some of which are also major tourist attractions (e.g., Dachstein Rieseneishöhle and Eisriesenwelt in the Austrian Alps).

Cave ice deposits are potentially important archives of local and regional paleoenvironmental change, as shown, for example, by studies of pollen preserved in cave ice^6–8^. Pits open to the surface act as natural snow gauges and their mass balance reflects long-term variations in solid (winter) precipitation modulated by winter temperature^9^. Previous studies have shown that robust chronostratigraphies can be established in firn and ice deposits of such sag-type caves using radiocarbon dating of organic macro remains. These studies include the dating of plant remains or insects that passively or actively entered the cave and became embedded in the ice. Studies of ice stratigraphies in such sag-type caves in the Austrian^10,11^, Swiss^12,13^ and Slovenian part^14^ of the European Alps, the Spanish Pyrenees^15^, and Idaho, USA^16^, have revealed semi-continuous records spanning the second half of the Holocene. Occasionally, additional techniques such as tritium or ^210^Pb have been used to constrain the age of the younger part of such ice deposits^12,17^.

In contrast, congelation ice deposits formed far from cave entrances are often devoid of organic macroremains. In ice caves with multiple entrances at different elevations, outside air only enters through the lower entrance(s) during the cold season, when the surface outside the cave is commonly snow-covered. Hence, chances are low that plant remains or insects find their way into the cave during winter and become embedded in ice. During the warm (plant growth) season, there is a strong outflow of cave air through the lower entrance(s) preventing plant remains or insects from entering the cave. Unless there are exceptional circumstances, such as the preservation of bat mummies^18,19^, congelation ice is typically much cleaner than firn ice in sag-type single-entrance caves and only few studies made attempts to date the former deposits despite their wide-spread occurrence.

May et al.^20^ studied a 7.3 m-thick deposit of congelation ice in Eisriesenwelt (Austria). Attempts to extract and radiocarbon-date micro amounts of particulate organic carbon from the deeper ice yielded a range of ages, likely reflecting the non-uniform source of this type of organic matter. Kern et al.^17,21^ examined a similar ice deposit in Dachstein-Mammuthöhle, also located in the Austrian Alps. Carbonaceous matter from different layers of this ice were subjected to stepped combustion heating prior to graphitization and radiocarbon analysis. Using the results of the 400°C combustion step, the authors obtained raw radiocarbon ages which were corrected for the input of aged organic carbon using age constraints obtained from tritium measurements. Linear extrapolation suggested that the bottom ice was probably deposited during the mid-5th millennium BP^17^.

An alternative approach to provide constraints on the internal age structure of congelation ice is dating of cryogenically formed carbonates. These minerals, mostly low-Mg calcite, occur as sub-millimeter size crystals and aggregates thereof embedded in layered ice, resembling dust layers^22^. Referred to as fine crystalline (or fine grained) cryogenic cave carbonates (fine CCC for short hereafter), these minerals have a distinct stable carbon and oxygen isotope composition consistent with strong degassing of carbon dioxide and crystallization of calcium carbonate as a consequence of fairly rapid freezing of a water film on ice^23^. Fine CCC are distinct from typically much larger crystals and crystal aggregates that form in pools on the ice, known as coarse CCC^22^. As fine CCC form at the same time as the ice, radiometric dating them would provide direct constraints on the age of the enclosing ice. A first attempt to constrain the age of fine CCC using radiocarbon techniques was made in two caves in the Canadian Arctic^24^ and we are unaware of more detailed follow-up studies exploring radiocarbon systematics of fine CCC. In addition to radiocarbon, there is a potentially attractive alternative method based on the ^230^Th disequilibrium, which has been widely used to date coarse CCC^25,26^. Dating fine CCC using this method, however, is challenging. The reason is the low ^230^Th/^232^Th ratio, reflecting the abundance of detrital Th, relative to the very low abundance of radiogenic ^230^Th in these commonly young samples. The only study that successfully applied this method to fine CCC was presented by Donner et al.^27^ who examined a small deposit of fine CCC and associated cryogenic sulphate minerals in a cave in NE Greenland.

These studies show that establishing chronologies of congelation ice deposits remains a difficult task. Here, we investigate the age structure of stratified congelation ice in Eisriesenwelt to gain first insights into the age and long-term dynamics of a large ice cave and tourist hotspot. Given the lack of organic macroremains, we applied radiocarbon and ^230^Th dating to CCC-bearing layers to obtain a robust age model, the first of its kind in such an Alpine ice cave.

## Study site

**Figure 1:**
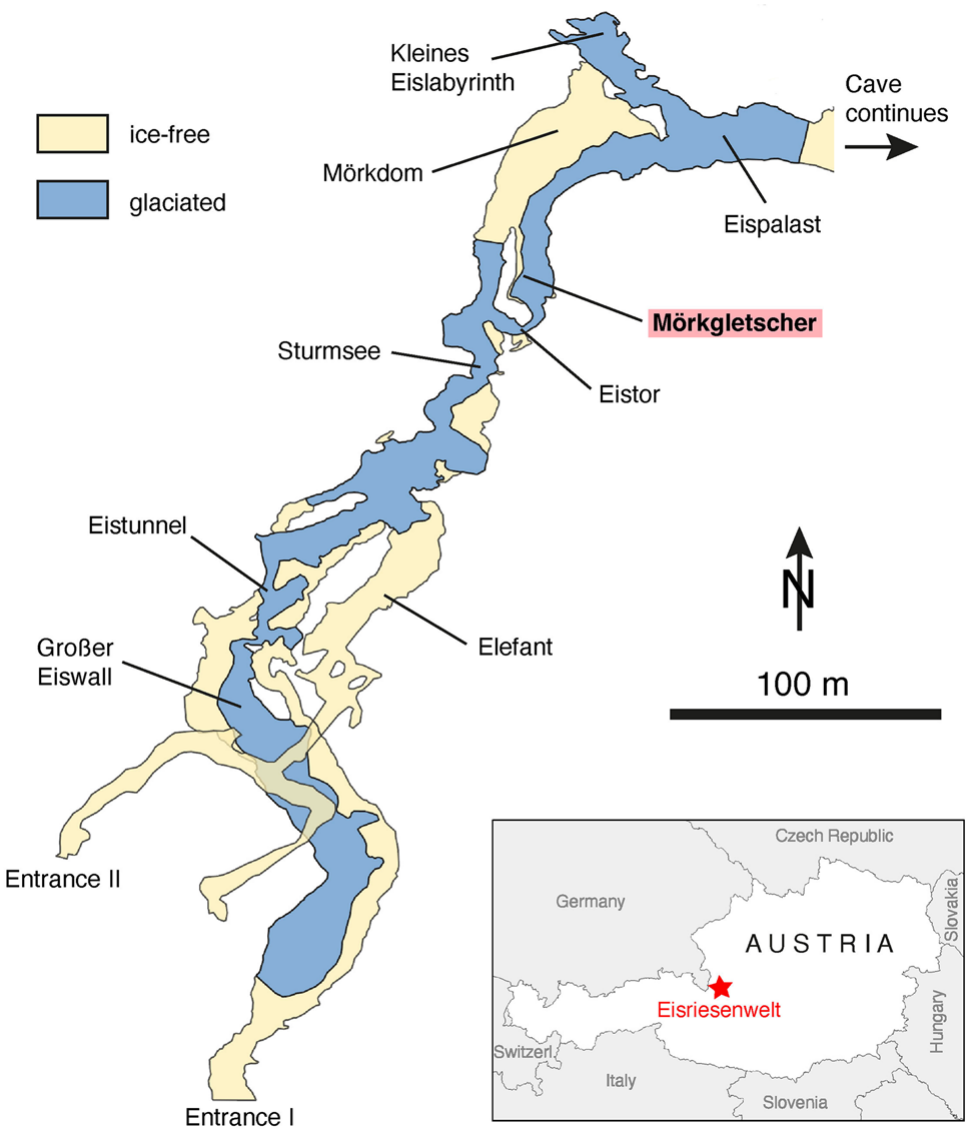
Simplified plan view of the frontal part of Eisriesenwelt showing the ice distribution, the study site Mörkgletscher, as well as other sites in this cave mentioned in the text. Map was produced using Adobe Illustrator (v27.2, www.adobe.com).

Eisriesenwelt is an extensive labyrinthic system of almost 40 km of passages in the western part of the Tennengebirge, a karst massif in the Northern Calcareous Alps of Austria (47° 30′ 40′′ N, 13° 11′ 35′′ E, Fig. 1). The main (lower) entrance opens in a cliff at an elevation of 1641 m a.s.l.; a much smaller, second (lower) entrance is located at 1838 m a.s.l. The upper entrance (possible multiple entrances) are likely located in dolines on the plateau of the Tennengebirge some 500 m above the main galleries and are not fully explored yet. As a result of this cave geometry, cold winter air enters through the lower entrances, and ascends through the cave’s passageways, eventually exiting on the plateau (chimney effect). This influx of winter air leads to a strong cooling of the first 550 m behind the main lower entrance and the preservation of congelation ice, covering an area of approximately 11,000 m^2^. In late spring, the atmospheric dynamics in the cave are reversed, as the cave air is cooler and denser compared to the warmer outside air, resulting in a strong, cold cave wind emanating from the lower entrances.

The main ice-forming season is spring and early summer, when the snow melts above the cave and this cold water seeps into the strongly undercooled frontal part of the cave. In contrast, ice sublimation typically occurs in winter by the fairly dry and cold air entering the cave. Minor ablation also occurs at the end of summer, particularly close to the inner border of the ice-bearing part. A large hall known as Eispalast (Fig. 1) is the demarcation point for year-round ice presence in the cave and is characterized by minimal seasonal temperature fluctuations when compared to the entirety of the ice-bearing portion of the cave^28^. Microclimatic measurements performed at the Eispalast during the period from December 2007 to November 2008 revealed that the mean cave air temperature in summer was approximately 0.0 °C, while the mean winter temperature registered at − 1.1 °C^29^. Deeper into the cave, only localized seasonal ice formations are observed, particularly during anomalously cold winters.

Eisriesenwelt offers spectacular exposures of the ice stratigraphy revealing well-bedded ice with varying amounts of air inclusions, but without organic macroremains. Hence, the age of this ice is largely unknown. The first attempt to date this ice was made half a century ago by studying pollen. Four ice samples from Mörkgletscher and Großer Eiswall (Fig. 1) yielded pollen spectra that included pollen of cultivated plants and were interpreted as probably formed during the Little Ice Age^30^. May et al.^20^ examined a core drilled into the floor ice of Eispalast. An estimate based on radiocarbon dating of particulate organic matter suggests a basal ice age of around 5000 yr BP, albeit with a large uncertainty.

**Figure 2:**
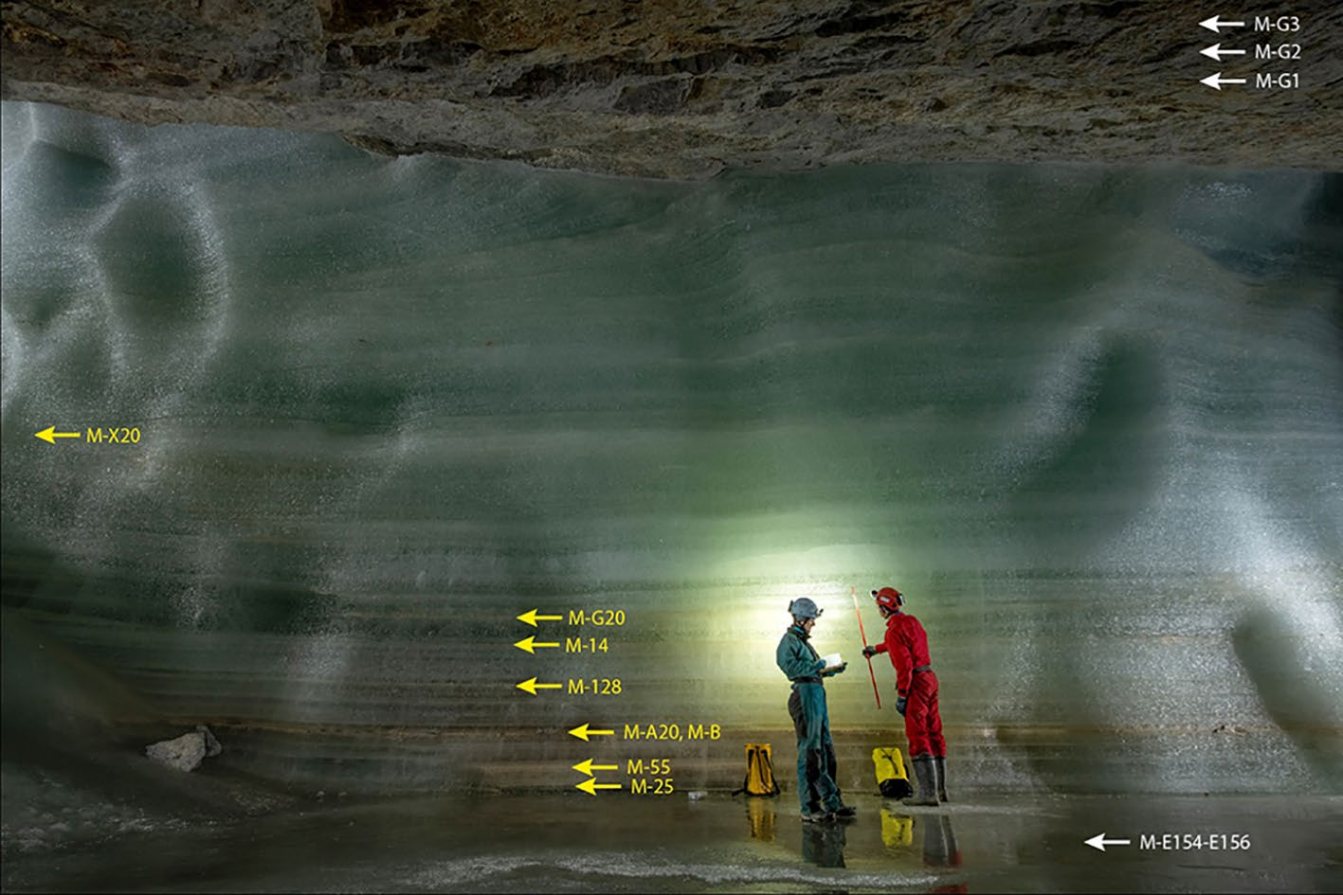
Exposure of stratified congelation ice at Mörkgletscher. CCC-bearing “dust” layers in the lower third of the cliff stand out because of their light brown color. Sampled CCC layers are labelled, whereby the lowermost sample (M-E154-E156) was taken from a core drilled vertically into the ice floor. The three uppermost samples (M-G1 to M-G3) were obtained from laterally discontinuous CCC layers on the opposite side of the ice cliff and stratigraphically above the highest ice shown on this picture. Photo courtesy Robbie Shone.

Exposures at several ice cliffs in Eisriesenwelt show the presence of fine CCC, most prominently at Mörkgletscher (Fig. 2). This 8 m high cliff (which extends higher up on the back side and comprises about 11.7 m of total ice exposure) shows several white to light brown layers in the lower part consisting of fine CCC, while the ice above is conspicuously cleaner and lacks CCC layers. These “dust layers” can be traced up to several meters along the ice cliff and pinch out laterally. In some of these layers angular blocks of limestone are intercalated. Based on an earlier study^31^, we systematically sampled the different CCC-bearing layers in this exposure, ranging in thickness from about 1 to several mm. In addition, we sampled thin CCC-lenses in the ice on the top of this cliff (on the back side) and extended the 11.7 m-long profile downward by coring the ice floor in front of the cliff (total almost 14 m) Finally, we also analyzed CCC extracted from ice drilled in Eispalast and from an exposure in Eistunnel (Fig. 1).

## Results and discussion

### CCC mineralogy and petrography

All samples consist of calcite. This also applies to two samples transported in cold condition to the laboratory and examined by XRD (see Methods). No evidence of precursor phases such as ikaite was found.

Under the microscope the samples are composed of > 99% euhedral calcite crystals and aggregates thereof. Granulometric measurements show median grain sizes between 44 and 199 µm (Table 1). The crystals show a homogeneous whitish color in reflected light. Impurities, i.e., detrital grains derived from the host limestone, are rare and easy to recognize based on their color (commonly light red to yellow) and anhedral shape.

**Table 1:** Grain size, radiocarbon and stable isotope data of CCC samples from Mörkgletscher (arranged according to depth, where 0 cm is the base of the ice in a drill core 1.9 m below the ice floor), as well as of two samples of experimentally formed CCC from Eispalast (L1-1, L2-2) and two CCC samples from Eispalast (F320) and Eistunnel (ETB).

Sample	Distance from base (cm)	Grain size (median, µm)	Lab no	Age (BP)	Error (±)	Age_corr_ (BP)	Age_corr_ (cal BP) 2 sigma	δ^13^C (‰)	δ^18^O (‰)
M-G3a*	1179	54	45,085	1369	19	948	918-792	9.51	− 9.35
M-G3b*	1179	54	45,780	1472	21	1051	1052-918		
M-G2	1149	56	45,084	1293	20	872	901-695	11.36	− 7.43
M-G1a*	1069	199	45,083	921	18	500	544-505	11.06	− 7.20
M-G1b*	1069	199	45,779	1012	18	591	646-542		
M-X20	487	44	43,296	1755	27	1334	1303-1177	13.25	− 5.35
M-G20	365	55	52,759	3004	26	2583	2760-2521	12.31	− 5.77
M-14	338		38,062	2480	22	2059	2105-1934	10.41	− 7.53
M-128	297	121	39,708	2501	26	2080	2124-1943	10.78	− 6.96
M-B**	247		38,060	3311	32	2890	3158-2885	10.93	− 6.32
M-A20**	247	58	43,295	2920	22	2499	2725-2471	10.60	− 6.59
M-55	224	40	43,293	3292	45	2871	3155-2868	9.81	− 7.21
M-25	194	54	39,707	3105	29	2684	2852-2749	9.69	− 6.87
M-E154-156a*	36		45,086	3363	23	2942	3207-2969	10.78	− 6.75
M-E154-156b*	36		45,781	3517	24	3096	3378-3226		
L1-1	–		47,410	356	20			2.32	− 18.50
L2-2	–		50,858	511	19			− 1.19	− 19.99
F320			45,088	3955	23	3534	3898-3699	9.04	− 8.62
ETB			43,299	1046	20	625	653-553	15.50	− 5.24

Three types of radiocarbon data are reported: conventional, corrected for the dead carbon effect, and corrected for the dead carbon effect and calibrated to calendar ages (see text).

*Duplicates analyzed from the same sample. **Taken laterally from the same CCC layer.

**Figure 3:**
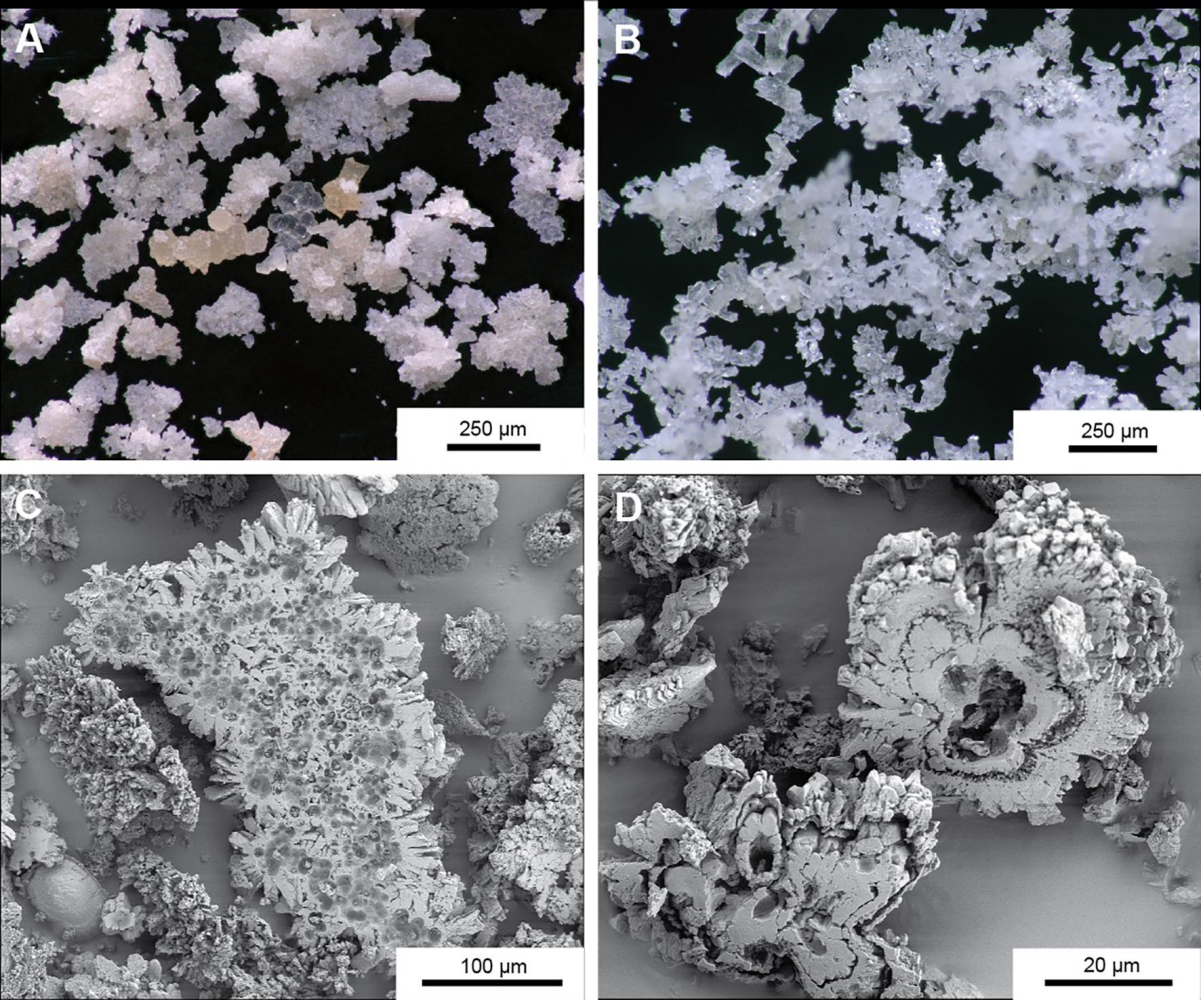
Microscopic appearance of fine CCC from Mörkgletscher. (**a**) and (**b**) are optical images of aggregates of euhedral calcite crystals showing a variety of particle shapes. (**c**) and (**d**) are SEM images of rafts and spheroids, respectively, both characterized by a conspicuously flat side where these particles touched the ice during growth.

Optical microscopy and SEM observations revealed a range of particle shapes. The most abundant type are crystal aggregates showing a radial arrangement of intergrown crystals (Fig. 3). These aggregates are often asymmetrical in structure and show a conspicuously flat upper side and a lower side covered with crystal tips. The upper side sometimes shows holes with a diameter of 5–15 µm. The second most abundant type are skeletal crystals or skeletal crystal aggregates. These show a very high microporosity. There are all transitions from crystal aggregates to skeletal crystals. Finally, there are (hemi)spherulitic aggregates showing a concentric shell structure. These particles often have a flat upper surface and are partly porous.

### CCC stable isotope composition

**Figure 4:**
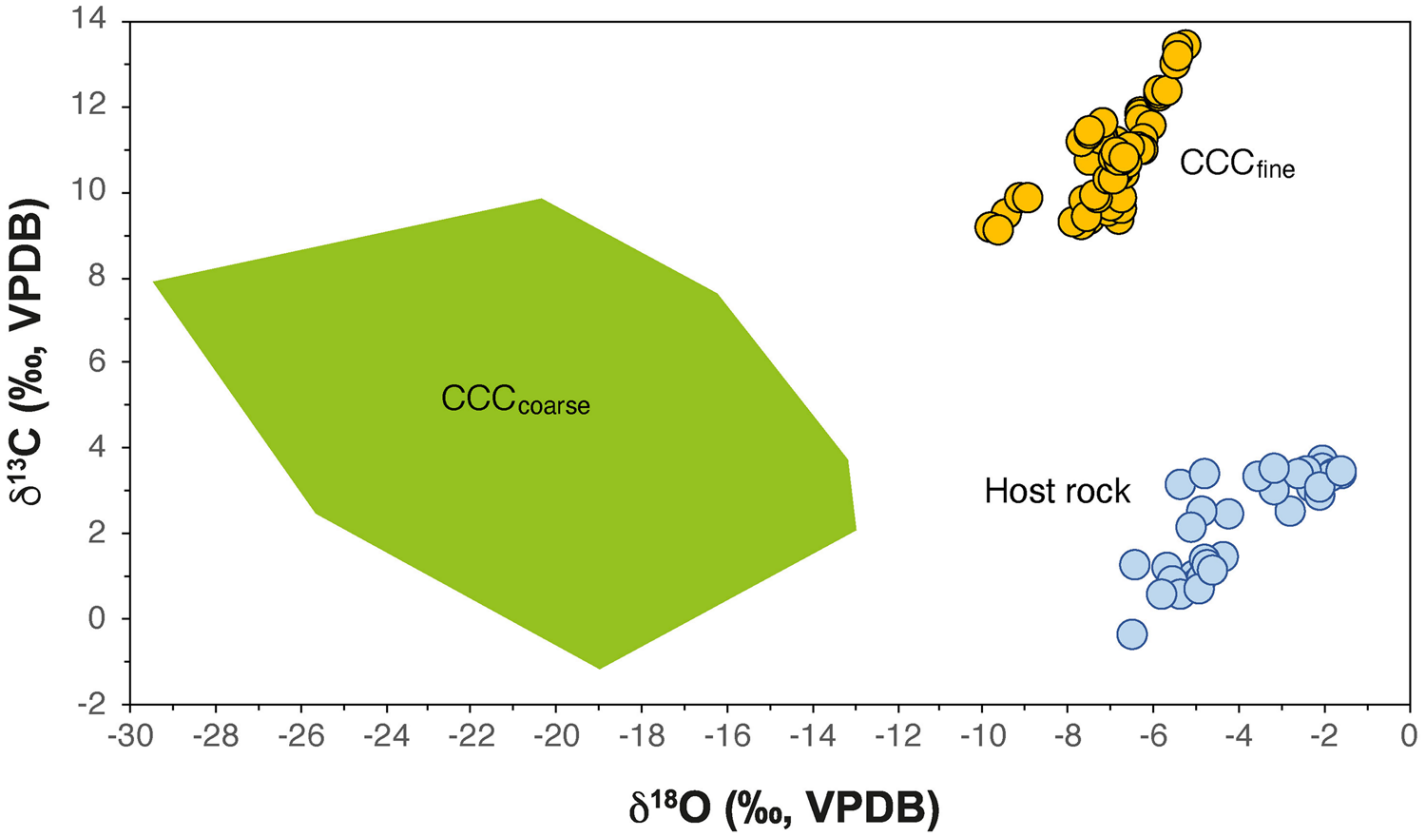
Carbon and oxygen isotopic composition of fine CCC from Mörkgletscher (n = 66) compared to Dachstein limestone (n = 38), the host rock of the cave (host rock data^31^). Also shown is the stable isotopic composition of coarse grained CCC from inner and currently ice-free parts of Eisriesenwelt (unpublished data by the authors).

Fine CCC from Mörkgletscher are characterized by highly positive δ^13^C values and moderately negative δ^18^O values (Fig. 4, Table 1). δ^13^C values reach as high as + 13.5‰. Aliquots measured of each CCC sample commonly show a tight clustering with only a few tenths of permil variability in both C and O isotopes; a few samples yielded a slightly larger variability of up to about 1.5‰ in δ^13^C and up to about 0.5‰ in δ^18^O. The C and O isotope data are linearly correlated (r^2^ = 0.52) with a positive slope of 0.83 (Fig. 4). These isotopic values are distinct from both calcite derived from weathering of the host rock limestone and from coarse grained CCC which are present in interior (and currently ice-free) parts of this cave and which document episodes of much more extensive cave glaciations prior to the Holocene (ref.^32^ and unpublished data by the authors).

### Radiocarbon results

**Figure 5:**
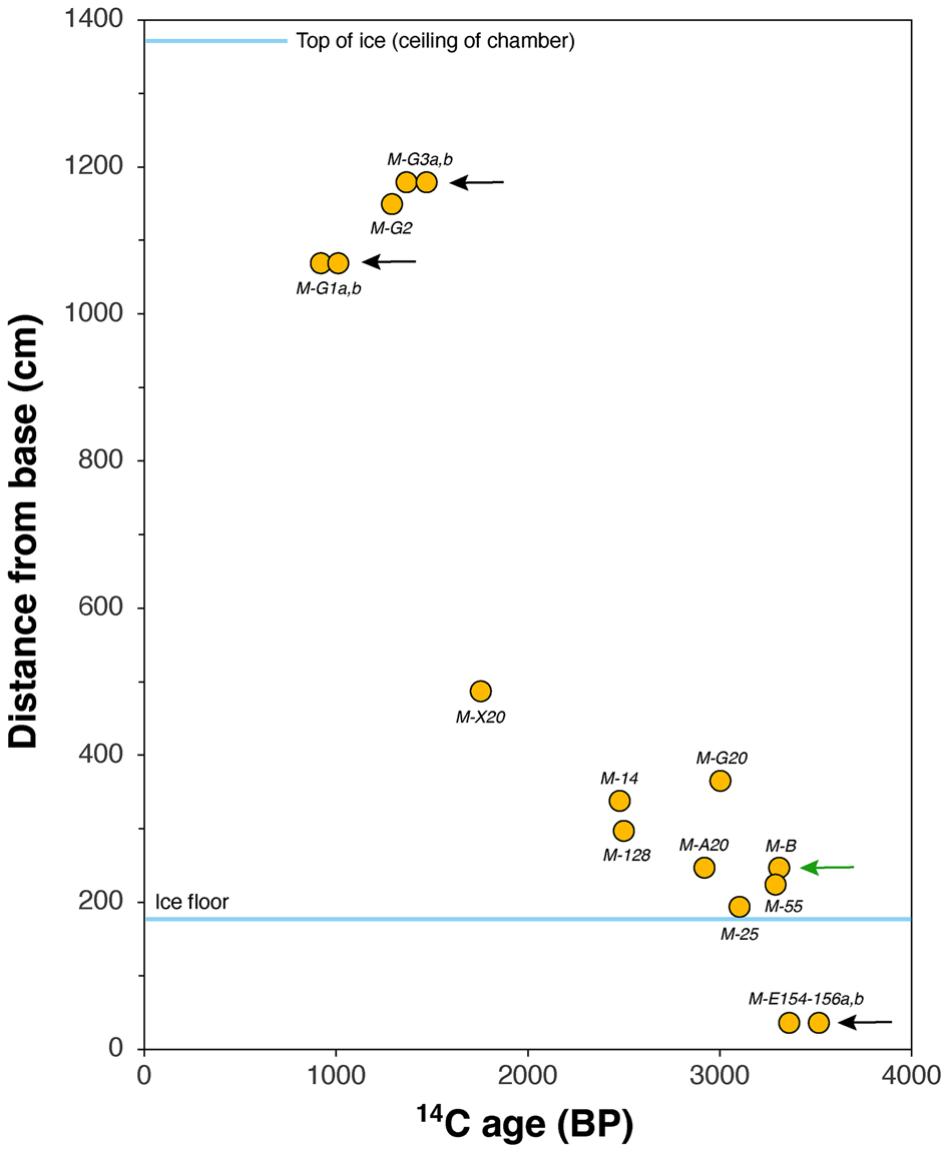
Conventional radiocarbon ages of fine CCC from the Mörkgletscher ice cliff plotted against ice thickness. The analytical uncertainty of the measurements is well within the size of the symbols. Black arrows indicate CCC layers where duplicate samples were analyzed. The green arrow marks two samples taken laterally within a CCC layer.

15 CCC samples from Mörkgletscher were analyzed for radiocarbon and the resulting conventional ages range from 921 to 3517 BP (Table 1). There is no relationship between ^14^C ages and δ^13^C values (r^2^ = 0.0002) suggesting that there is no significant detrital contamination by radiocarbon-free host rock particles characterized by much lower δ^13^C values (Fig. 4). Replicates of samples from three CCC layers yielded conventional ages that agree within 4.4, 7.0 and 9.0% and two samples taken laterally within one CCC layer deviate by 12% (Table 1). Plotted against depth, the conventional ages decrease from the bottom of the ice section up, with the uppermost samples being approximately 2000–2500 ^14^C years younger than the deepest ones (Fig. 5).

DIC of a sample (ERW-B) taken from the water coming from the Alphorn shaft yielded 95.2 ± 0.5% pmC, which indicates a low dead carbon effect (4.8%) and a small reservoir age (395 ± 42 ^14^C yr). CCC formed experimentally in small pools in Eispalast^33^ yielded conventional radiocarbon ages of 356 ± 20 BP (sample L1-1, using water from the Alphorn shaft) and 511 ± 19 BP (samples L2-2, using water from the Elefant site—Fig. 1, Table 1).

### ^230^Th results

Three CCC samples were analyzed for ^230^Th. Sample M-G1 from near the top of the ice section yielded an age of 953 ± 303 yr BP. Sample M-A20 from the lower part of the cliff and sample M-E154-156 taken from a drill core at the foot of the cliff yielded ages of 2262 ± 4346 and 3737 ± 1954 yr BP, respectively, which are in stratigraphic order but very imprecise due to the large Th correction (Table 2).

**Table 2:** ^230^Th ages of CCC samples from Mörkgletscher.

Sample number	^238^U (ppb)		^232^Th (ppt)		^230^Th/^232^Th (atomic × 10^–6^)		δ^234^U* (measured)		^230^Th/^238^U (activity)		^230^Th Age (yr) (uncorrected)		^230^Th Age (yr) (corrected)		δ^234^U_Initial_** (corrected)		^230^Th Age (yr BP)*** (corrected )	
M-G1	136.7	± 0.4	2831	± 57.0	15	± 0.7	439.3	± 4.8	0.0189	± 0.0008	1443	± 63.8	1024	± 303.2	441	± 4.8	953	± 303
M-A20	283.6	± 0.6	85,096	± 1711	6	± 0	460.7	± 2.2	0.1094	± 0.0010	8453	± 78	2335	± 4346	464	± 6	2262	± 4346
M-E154-E156	280.5	± 0.6	37,681	± 756.9	10	± 0.2	429.2	± 3.7	0.0837	± 0.0008	6565	± 67.7	3808	± 1954.3	434	± 4.4	3737	± 1954

U decay constants: λ_238_ = 1.55125×10^-10^ (Jaffey et al.^55^) and λ_234_ = 2.82206×10^-6^ ref.^54^. Th decay constant: λ_230_ = 9.1705×10^-6^ ref.^54^.

*δ^234^U = ([^234^U/^238^U]_activity_ – 1)x1000. **δ^234^U_initial_ was calculated based on ^230^Th age (T), i.e., δ^234^U_initial_ = δ^234^U_measured_ x $\text e^{{\lambda_{234}}\times \text T}$. Corrected ^230^Th ages assume the initial ^230^Th/^232^Th atomic ratio of 4.4 ± 2.2 ×10^-6^. Those are the values for a material at secular equilibrium, with the bulk earth ^232^Th/^238^U value of 3.8. The errors are arbitrarily assumed to be 50%. ***BP stands for “Before Present” where the “Present” is defined as the year 1950 A.D.

### Origin of the CCC layers and the ice cliff

Several lines of observations show that the discontinuous “dust layers” at Mörkgletscher are deposits of fine CCC with only tiny traces of other, i.e., detrital (limestone) particles. These observations include the euhedral shape and the stable isotopic composition of these carbonate crystals and crystal aggregates. The stable isotopic composition is not only distinct from that of the local limestone, but also consistent with fine CCC extracted from an ice core drilled in the Eispalast^20^ and fine CCC found on top of modern ice surfaces in this cave (unpublished data by the authors). The C isotope values overlap with fine CCC from ice caves in Romania^34^ and the Canadian Arctic^35^, but the O isotope values are shifted towards higher (Romania) and lower values (Canada), reflecting the overall δ^18^O values of meteoric precipitation (higher and lower in Romania and Canada, respectively). These data suggest fairly rapid crystallization from a thin water film on ice surfaces. The abundance of skeletal crystals and spheroidal forms is consistent with this mode of formation.

A special feature of the CCC layers at Mörkgletscher is their varying thickness and their lateral discontinuity. Both indicate that the thicker layers in particular were not deposited in situ, but represent redeposition of CCC. Our own observations made in winter in the ice-bearing part of Eisriesenwelt showed that fine CCC are being released from ice surfaces due to sublimation, forming tails of white to light brown powders. Similar observations were reported from ice caves in the Canadian Arctic where sublimation released CCC disseminated in ice, forming CCC deposits up to 5 cm thick^24^. These fine powders can be transported by the cave wind, which in Eisriesenwelt can reach more than 10 m/s on cold days in areas of narrow cross sections. It is interesting to note that cavers who explored and surveyed the cave about a century ago reported “flour-like dust often in finger-thick layers” at a narrow squeeze known as Sturmsee^36^ (this narrow passage had to be overcome by crawling and was later artificially widened; Fig. 1). These deposits were likely re-worked fine CCC deposited in the lee of this formerly strongly ventilated squeeze. A similar setting likely gave rise to the CCC layers at Mörkgletscher, located in the lee of another squeeze called Eistor (Fig. 1). Back in 1913, the first explorers had to crawl through a narrow gap between the ice and the rock ceiling to enter the hall called Mörkdom (Fig. 1) which hosts Mörkgletscher^37^. It is thus likely that these fine CCC had formed slightly further west (i.e., closer to the cave entrance) and became released from the ice by sublimation as a result of cold winter air being drawn into the cave through the main entrance. This hypothesis could also explain the fact that the CCC layers pinch out laterally towards the east, i.e., leeward from the former Eistor squeeze. Angular blocks of limestone embedded in some of the thickest CCC layers (but absent in clean ice above) represent breakdown from the ceiling, likely as a result of strong cooling and frost shattering. The thicker CCC layers therefore likely represent microhiatuses reflecting anomalously cold winter conditions giving rise to strong sublimation and short-distance eolian transportation of fine CCC and redeposition behind narrow passages followed by burial by younger layers of ice. There is no evidence, however, that the thicker CCC layers mark major (long lasting) episodes of ice retreat. The presence of the CCC layers marking microhiatuses therefore indicates that the mean rate of ice accumulation in the lower part of the profile was likely lower compared to the middle and upper part, where CCC layers are rare, thinner and can only be traced laterally for less than a few meters.

The ice cliff of Mörkgletscher did not exist in 1913, when the ice body extended all the way to the opposite cave wall. By about 1925, a 2 m-wide gap had opened due to ice sublimation^37^, which since then has widened to almost 8 m, exposing the internal structure of this floor ice and its CCC layers. The average rate of ice wall retreat due to sublimation during the cold season (plus a smaller contribution of summer melting) was about 6 cm per year.

### Radiocarbon and ^230^Th constraints on the age of the CCC layers

Although the (thicker) CCC layers in the lower part of the ice section likely mark microhiatuses, they are nevertheless the key to obtaining quantitative information on the age of the surrounding ice. The purity of the CCC samples in terms of detrital contamination and the replicated samples are strong arguments that the overall decrease in conventional radiocarbon ages with increasing distance from the base of the section reflects an age gradient of approximately 2400 ^14^C years, using the youngest radiocarbon age of each layer (Fig. 5). To constrain the timing of the start and end of ice accumulation, the radiocarbon dates must be corrected for the effect of dead carbon, followed by calibration to calendar years.

**Figure 6:**
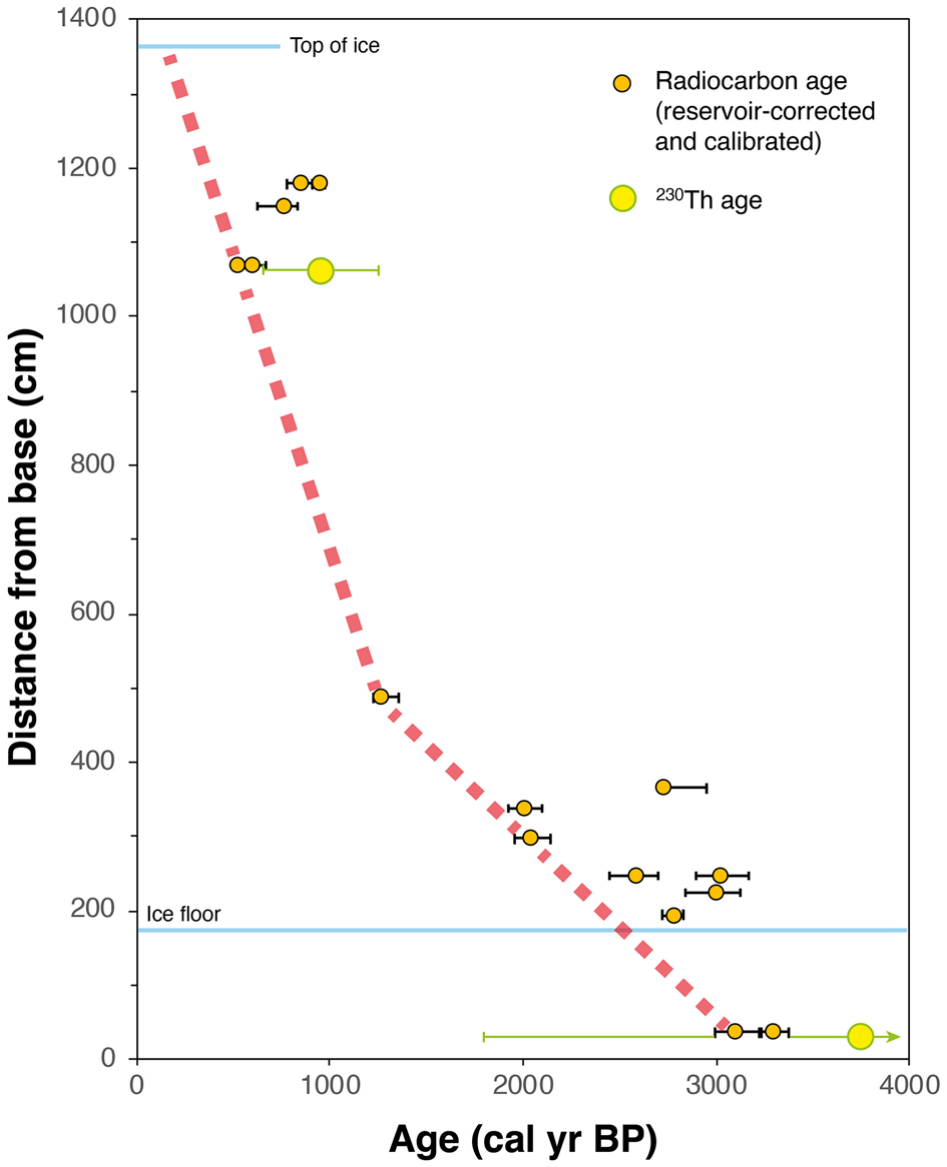
Radiocarbon ages of fine CCC samples from the Mörkgletscher ice cliff corrected for a constant reservoir age of 421 ± 17 ^14^C years and calibrated, showing median ages and associated 2σ uncertainties. The two yellow-green data points are ^230^Th ages with their 2σ uncertainties. The thick dashed red line is the proposed depth-age model based on the youngest ages of each sampled CCC layer.

The dead carbon effect was assessed using radiocarbon analyses of modern CCC and DIC and compared to less precise constraints provided by ^230^Th dating. The radiocarbon dates of two modern CCC samples formed in the nearby Eispalast (356 ± 20 BP and 511 ± 19 BP) differ somewhat, reflecting the different water sources used for the two freezing experiments^33^. Such small radiocarbon variabilities of DIC are not uncommon in caves^38,39^. The value of DIC of one of the waters (395 ± 42 BP) is consistent with the CCC formed from it (356 ± 20 BP; the second water was not analyzed). We used the mean of all three measurements and its standard error (421 ± 17 BP) to correct the radiocarbon dates of CCC samples from Mörkgletscher. This approach assumes that the dead carbon effect has remained constant during the past few millennia. This appears justified because major changes in the composition and bioproductivity of the thin and patchy soil present above this part of the cave and of water–rock interactions in the vadose zone above the cave are unlikely in this time frame. This also includes land use changes which can be ruled out for the steep and rocky terrain above the cave. The resulting dead carbon-corrected dates were then converted to calendar ages using IntCal20^40^ (Fig. 6). The final ages are plotted on Fig. 6 together with the proposed age model which is constrained by the youngest ages of each dated CCC layer. The rationale behind this is the following: Several processes may result in calibrated radiocarbon ages being variably older than the “true” age. These include (a) contamination by detrital carbonate, (b) a higher dead carbon fraction, and/or (c) reworking of CCC derived from older ice strata. On the other hand, processes that would result in anomalously young CCC ages such as contamination by younger CCC or organic matter can be ruled out. The proposed linear age model is constrained by the youngest ages of each CCC layer following the rationale outlined above. A more accurate and precise depth-age model is currently not possible given the nature of the radiocarbon dates of such CCC samples (which represent maximum age estimates) and the limited number of CCC layers. The validity of our proposed age model, however, is confirmed in the upper part by a ^230^Th age whose 2σ uncertainty almost overlaps with the age model (Fig. 6). The ^230^Th age obtained from the deepest CCC sample is imprecise but still provides an independent age control, and it is consistent with the age model even within its 1σ uncertainty range (Fig. 6). The third ^230^Th age is unfortunately too imprecise to be used.The proposed depth-age relationship assumes that no melting had occurred at the base of this ice body. Basal melting would remove older ice and hence truncate the stratigraphic record. This process has been documented in some ice caves (e.g.^9,41^), but several lines of observations suggest that it does not play a significant role in Eisriesenwelt. (i) In all places in the cave where the contact between the basal ice and the rock or gravel beneath is well exposed, there is no macroscopic evidence of melting (based on observations during all seasons, but primarily during summer and autumn). If cave ice reaches the melting point it changes in crystal fabric resulting in a honeycomb-shaped structure (known as Wabeneis^42^). Such features were locally observed at the surface of ice stalagmites in late summer but were never observed in the basal ice. (ii) Ice temperature profiles obtained in Eispalast recorded negative temperatures year-round in the deepest ice layer at 7.3 m depth close to the base of the ice (unpublished data by the authors). Also, an earlier study of ice temperatures at Eispalast based on temperature profiles found no evidence of basal melting^29^. (iii) There is no evidence of measurable ice movement in this cave (due to basal sliding), even in parts where the ice forms steep slopes. This is based on decadal-long observations by the show cave personnel (pers. comm. A. Rettenbacher). If there had been ice movement, fortifications for the tourist trails drilled vertically into the ice slopes would have been deformed and/or tilted, which was not observed. This argues for the ice being permanently frozen to the rock or gravel substrate. In some exposures ice strata are inclined and change their dip laterally, mimicking folding. This structure, however, is not related to ice folding, but reflects the growth structure of large ice stalagmites and their variable internal stratification when exposed along an ice tunnel or cliff. In summary, there is no evidence of basal melting in Eisriesenwelt. During colder climate periods such as the Little Ice Age basal melting was thus even more unlikely.

### Implications for long-term changes of the cave ice mass balance

The proposed age model suggests that ice accumulation at Mörkgletscher commenced about 3.2 cal kyr BP and terminated at about 0.2 cal kyr BP. The age model suggests a mean accumulation rate of 0.25 cm per year during the first two millennia until about the thirteenth century AD, followed by a mean rate of about 0.79 cm per year in the subsequent six centuries. This overall increase in growth rate is consistent with the scarcity of CCC layers (and the lack of thick CCC layers) in the middle and upper part of the section. In contrast, the abundance of CCC layers in the lower part suggests repeated episodes of intensive ice sublimation (possibly also minor ablation), hence overall lower accumulation rates, but still an overall positive ice mass balance.

The onset of ice accumulation at Mörkgletscher around 3.2 kyr BP may have been related to an episode of glacier advance in the Alps, known as the Göschenen 1 advance and more recently called Iron Age Advance Period 1, starting around 3 kyr BP^43^. The change towards a higher mean accumulation rate during approximately the thirteenth century AD occurred at the onset of the Little Ice Age, which started in the Alps around 1260 AD and ended at about 1860 AD^44^. In addition, the end of the ice accumulation phase—based on extrapolation (Fig. 6)—falls at the end of the Little Ice Age. In the 1920s, the ice had started to retreat from the southern cave wall and already exposed part of the Mörkgletscher ice cliff, i.e. the mass balance in this inner part of the show cave had become negative by then. Since then, the ice cliff has retreated by about 6 cm per year.

We also tested the CCC-dating approach in two other parts of Eisriesenwelt and briefly report here preliminary results. First, we applied this approach to Eispalast (Fig. 1), where an ice core drilling had encountered 7.3 m of ice^20^. We re-drilled this ice body and sampled a CCC layer at 3.2 m depth (sample F320, 3955 ± 23 BP). Using the same correction for the dead carbon effect as applied above, the calibrated age suggests that this ice is not more than 3.9–3.7 cal kyr BP old (2σ range, Table 1). Whether the basal ice is about 5 millennia old as suggested by imprecise radiocarbon data of particulate organic matter^20^ remains an open question.

Secondly, we sampled fine CCC layers in a place called Eistunnel, which is a tunnel that was artificially cut through the ice in the middle part of the show cave in 1963 (Fig. 1; pers. comm. A. Rettenbacher) and which has since widened substantially by sublimation. Multiple layers of fine CCC are present in this ice whose gravel base is well exposed. A sample from a CCC layer about 45 cm above the ice base yielded a radiocarbon age of 1046 ± 20 BP (sample ETB). When corrected for the dead carbon effect the same way as the Mörkgletscher samples, the calibrated age is 653–553 cal yr BP (2σ range, Table 1), which suggests that this ice was likely formed between about the fourteenth and fifteenth century, assuming that no ice was lost due to basal melting. As detailed above for the Mörkgletscher site, basal melting does not play a significant role in this cave today. Given the more proximal location of Eistunnel with respect to the lower cave entrance, basal melting is even more unlikely there compared to Mörkgletscher. In essence, this CCC age falling into the Little Ice Age implies that the central (and likely also the frontal) part of the show cave may have been largely ice-free during medieval times. The observation that older ice is preserved in the inner part (Mörkgletscher, Eispalast) is in contrast to what would be expected from the point of maximum winter cooling (more pronounced in the near-entrance part). Part of this discrepancy may be explained by (i) more pronounced ice sublimation in the near-entrance part due to a greater temperature difference, (ii) the presence of large depressions in the inner part allowing thick and less vulnerable floor ice deposits to form compared to the more proximal parts that are characterized by steep slopes, and (iii) local ingress of water. Today, large ice stalagmites form in the cooler parts of the cave close to the entrance due to the drip water, while water entering the inner parts of the ice cave mostly forms sheet-like deposits (unless artificially diverted).

The fact that the ice at Mörkgletscher has been retreating for about a century suggests that the current warming has already penetrated deeper into the cave than during the Medieval Warm Period. In this context, it is important to mention that the cave’s microclimate has been artificially modified for about a century by closing the lower entrance with a door during the warm season (to prevent the cold cave air from escaping). Old surveys indeed shows more ice in Eisriesenwelt during the 1950s and 1960s compared to 1920^32^. Without this door, the summer cave temperatures would be higher, the near-entrance part of the show cave would most likely be ice-free (only seasonal ice) and the ice deposits in the inner ice parts would probably become increasingly vulnerable.

### Perspectives

Radiometric dating of congelation ice remains a challenging task, which ultimately limits attempts to unravel the full potential of this poorly understood paleoenvironmental archive.

Given the wide-spread lack of organic matter in this type of cave ice (and the challenge to constrain soil and karst reservoir effects), the use of fine CCC as a chronometer seems an attractive way forward. In terms of radiocarbon, two aspects are important. First, radiocarbon dates of CCC are maximum age estimates and the accuracy of the final age hinges on the magnitude of the local dead carbon effect. Secondly, fine CCC disseminated in ice and thin CCC layers likely formed together with the surrounding ice. In contrast, thicker CCC layers represent microhiatuses and require sublimation of pre-existing CCC-bearing ice followed by short-distance transportation by cave wind to accumulate in such a concentrated manner. The issue of the dead carbon effect could be tackled by a systematic DIC study of modern cave water, but we are unaware of such a kind of study in ice caves. Depending on the size of the cave, the thickness of the rock overburden, the types of water pathways, and the structure of the soil, it is likely that such a study would yield a range of values for the dead carbon effect, probably also on a seasonal scale. Applying such a modern average correction factor to millennia-old ice requires independent age control. This is why ^230^Th data of fine CCC would be highly valuable. Given their very large surface area, the delicate CCC crystals and crystal aggregates are inherently less pure with respect to detritally bound initial ^230^Th than regular speleothems such as stalagmites. Attempts to purify fine CCC samples by repeated washing in an ultrasonic bath and hand-picking of contaminant particles under a binocular were only partially successful as shown by the still very low ^230^Th/^232^Th ratios (Table 2) resulting in large corrections. A way to obtain more accurate dates of fine CCC is whole-sample dissolution and isochron methods in order to distinguish between isotope ratios of the detrital and the authigenic phases^45^. This requires extra laboratory effort and cannot be done for many samples, but would provide a few important anchor points against which a radiocarbon-based ice stratigraphy could be compared.

Finally, there is a novel radiometric technique emerging which utilizes the very rare noble gas ^39^Ar radioisotope, trapped in ice during its formation. The half-life of ^39^Ar is 269 years, permitting the direct dating of ice as old as about two millennia^46^. In a test study, 4.2–6.7 kg samples of ice from the base of two glaciers in the Tyrolian and the Swiss Alps were analyzed. Though the results are encouraging they are associated with substantial measurement uncertainties, e.g., 1126 + 1286/− 273 years in the case of the oldest of these samples, which involved counting 31 atoms in 20 h^47^. Five cave ice samples were analyzed for the abundance of ^39^Ar from Leupa Ice Cave located in the Julian Alps of northern Italy. The results also show large analytical uncertainties^48^. Although ^39^Ar measurements are currently performed by only few laboratories worldwide and are very time consuming, major improvements in count rate (F. Ritterbusch, pers. comm.) hold reasonably high promises that congelation ice of the common era can eventually be reliably dated using this technique.

## Conclusions

Radiocarbon and ^230^Th ages obtained on CCC interbedded with congelation ice suggest that the giant ice deposits in the inner part of Eisriesenwelt (Mörkgletscher) started to accumulate at the end of the 4th millennium, probably related to the Iron Age Advance Period 1 known from Alpine glacier records. A distinct increase in the average ice accumulation rate occurred around the thirteenth century AD, coinciding with the onset of the Little Ice Age. According to a linear extrapolation, the youngest ice this part of the cave formed at the end of the Little Ice Age. Data from a second ice exposure closer to the entrance yielded a basal ice age that also falls into the Little Ice Age. We therefore conclude that a significant part of the current ice volume of Eisriesenwelt originated in the cold centuries of the Little Ice Age, while the near-entrance and central parts of the cave were likely mostly ice-free during the warm Middle Ages. These observations provide an important long-term perspective to assess the resilience of this large ice cave in a warming climate.

## Methods

### Sampling

Sampling of CCC-rich layers at Mörkgletscher was accomplished using an ice screw drilled parallel to bedding into these impure ice layers and collecting the crushed ice in plastic bags. Short cores, 7.25 cm in diameter, were hand-drilled vertically at the base of the cliff using a Mark III Kovacs ice corer (https://kovacsicedrillingequipment.com). The cores reached a depth between 1.4 and 1.9 m before hitting rock. Some 10 m to the north, at the northern end of this cliff, an ice thickness of 2.9 m was obtained by steam-drilling, but no core was taken. In summary, up to almost14 m of ice are present at Mörkgletscher and a total of 11 CCC-bearing layers were sampled (Fig. 2). Four of these layers were sampled in duplicate.

Samples of CCC-bearing ice were allowed to melt in the laboratory. CCC were separated by settling in a beaker followed by drying at 50 °C. The mineralogical composition of CCC samples was analyzed using X-ray diffractometry (XRD). We also obtained two samples from the CCC layers at Mörkgletscher that were transported in cold conditions to the laboratory in Budapest (Hungary) where XRD analyses were performed to check for possible precursor carbonate minerals.

Water for radiocarbon measurements of dissolved inorganic carbon (DIC) was collected in 500 ml glass bottles leaving no head space. One sample (ERW-B) was taken from the water supply line of the cave which at that time was mainly sourced from the Alphorn shaft located about 780 m deeper into the cave from the Mörkgletscher. This sample was analyzed for the radiocarbon concentration of DIC using accelerator mass spectrometry (by Hydroisotop, Schweitenkirchen, Germany).

We also produced CCC experimentally in a side passage next to Eispalast (called Kleines Eislabyrinth—Fig. 1) by letting water freeze in holes cut into the floor ice using a chain saw. These experiments were designed to simulate the formation of pool-type subaqueous CCC and water from different places in the cave was used. The resulting CCC were fine grained^33^ and two bulk samples were analyzed for radiocarbon. CCC sample L1-1 was formed by freezing of water obtained from the Alphorn shaft, while water from a site called Elefant (Fig. 1) was used in a freezing experiment that led to the crystallization of CCC sample L2-2.

### Grain size and petrography

The size distribution of the CCC samples was determined using laser diffraction granulometry (Malvern Mastersizer 3000) with a detection range of 0.01–2000 μm. The morphology of CCC was examined using a digital microscope (Keyence VHX-5000) and a scanning electron microscope (SEM, Zeiss DSM 982 Gemini).

### Stable isotope measurements

Small aliquots (0.1–0.2 mg) of CCC were analyzed for their C and O isotopic composition using a Delta V plus isotope ratio mass spectrometer coupled with a Gasbench II carbonate preparation and purification device (Thermo Scientific). Samples were reacted with 100% orthophosphoric acid at 80 °C. The results are reported on the VPDB scale. The long-term analytical precision of the δ^13^C and δ^18^O values was 0.06‰ and 0.08‰, respectively (1σ)^49^.

### Radiocarbon measurements

For radiocarbon dating, CCC samples were examined under a binocular and rare grains of detrital origin (typically yellow to brown and angular compared to white and euhedral CCC grains) were removed by hand-picking using a needle. The purified aliquots were then prepared at Queen’s University of Belfast (Ireland). The samples were placed in a septa seal vial (exetainer) and an amount of 1% HCl calculated according to the weight of the sample was added to remove 25% of the surface carbonate. The samples were then washed and rinsed using deionized water and hydrolyzed with phosphoric acid (H_3_PO_4_) in their exetainers on a heating block at 90 °C to evolve CO_2_^50^. The CO_2_ was converted to graphite on an iron catalyst using the zinc reduction method^51^. The ^14^C/^12^C and ^13^C/^12^C ratios were measured by accelerator mass spectrometry (AMS) at the ^14^CHRONO Centre, Queen’s University Belfast. The sample ^14^C/^12^C ratio was background corrected and normalized to the HOXII standard (SRM 4990C; National Institute of Standards and Technology). The radiocarbon ages and F^14^C were corrected for isotope fractionation using the AMS measured δ^13^C which accounts for both natural and machine fractionation. The radiocarbon age and one standard deviation were calculated using the Libby half-life of 5568 yr following the methods of Stuiver and Polach^52^.

### ^230^Th dating

A second set of aliquots of three CCC samples was cleaned using repeated ultrasonic treatments and hand-picking of rare detrital grains under a microscope using a needle and subjected to ^230^Th dating. The analyses were performed at the Isotope Laboratory of Xi’an Jiaotong University, using standard chemistry procedures to separate U and Th^53^. U and Th isotopes were measured using a multi-collector inductively coupled plasma mass spectrometer (ThermoFinnigan Neptune Plus) equipped with a MasCom multiplier behind the retarding potential quadrupole in peak-jumping mode. Instrumentation and standardization are described in ref.^53^. ^230^Th ages were calculated using decay constants of Cheng et al.^54^ and the uncertainties at the 2σ level include corrections for blanks, multiplier dark noise, abundance sensitivity and contents of nuclides in the spike solution. ^230^Th ages are reported in years BP, i.e., before the year 1950 AD.

## Data Availability

All data generated or analyzed during this study are included in this publication or are available from the first author upon request.
